# Detection of Rift Valley Fever Virus in *Aedes* (*Aedimorphus*) *durbanensis,* South Africa

**DOI:** 10.3390/pathogens11020125

**Published:** 2022-01-21

**Authors:** Carien van den Bergh, Peter N. Thompson, Robert Swanepoel, Antonio P. G. Almeida, Janusz T. Paweska, Petrus Jansen van Vuren, William C. Wilson, Alan Kemp, Estelle H. Venter

**Affiliations:** 1 Department of Veterinary Tropical Diseases, Faculty of Veterinary Science, University of Pretoria, Onderstepoort 0110, South Africa; bobswanepoel@gmail.com (R.S.); estelle.venter@jcu.edu.au (E.H.V.); 2 Department of Production Animal Studies, Faculty of Veterinary Science, University of Pretoria, Pretoria 0002, South Africa; peter.thompson@up.ac.za; 3 Unidade de Parasitologia Medica, Global Health and Tropical Medicine, Universidade Nova Lisboa, 1365-008 Lisboa, Portugal; PAlmeida@ihmt.unl.pt; 4 Centre for Emerging Zoonotic and Parasitic Diseases, National Institute for Communicable Diseases of the National Health Laboratory Service, Sandringham, Johannesburg 2192, South Africa; januszp@nicd.ac.za (J.T.P.); Petrus.Jansenvanvuren@csiro.au (P.J.v.V.); alan.kemp24@gmail.com (A.K.); 5 Department of Medical Virology, Faculty of Health Sciences, University of Pretoria, Pretoria 0002, South Africa; 6 School of Pathology, Faculty of Health Sciences, University of the Witwatersrand, Johannesburg 2000, South Africa; 7 Australian Centre for Disease Preparedness, CSIRO-Health and Biosecurity, Geelong, VIC 3220, Australia; 8 National Bio and Agro-Defense Facility, Agricultural Research Service, United States Department of Agriculture, Manhattan, KS 66502, USA; william.wilson2@usda.gov; 9 College of Public Health, Medical and Veterinary Sciences, James Cook University, Townsville, QLD 4811, Australia

**Keywords:** *Aedes (Aedimorphus) durbanensis*, transmission, mosquito vector, Rift Valley fever virus

## Abstract

Rift Valley fever virus (RVFV) is a mosquito-borne, zoonotic phlebovirus-causing disease in domestic ruminants and humans in Africa, the Arabian Peninsula and some Indian Ocean islands. Outbreaks, characterized by abortion storms and a high morbidity rate in newborn animals, occur after heavy and prolonged rainfalls favouring the breeding of mosquitoes. However, the identity of the important mosquito vectors of RVFV is poorly known in most areas. Mosquitoes collected in the Ndumo area of tropical north-eastern KwaZulu-Natal (KZN), South Africa, were tested for RVFV nucleic acid using RT-PCR. The virus was detected in a single pool of unfed *Aedes* (*Aedimorphus*) *durbanensis*, indicating that this seasonally abundant mosquito species could serve as a vector in this area of endemic RVFV circulation. Phylogenetic analysis indicated the identified virus is closely related to two isolates from the earliest outbreaks, which occurred in central South Africa more than 60 years ago, indicating long-term endemicity in the region. Further research is required to understand the eco-epidemiology of RVFV and the vectors responsible for its circulation in the eastern tropical coastal region of southern Africa.

## 1. Introduction

Rift Valley fever virus (RVFV) (order *Bunyavirales*, family *Phenuiviridae*, genus *Phlebovirus*) [1] causes severe, intermittent and sporadic outbreaks of Rift Valley fever (RVF) in domestic ruminants in Africa, the Arabian Peninsula, Comoros, Mayotte and Madagascar [2,3,4]. This is a mosquito-borne disease usually recognized by the onset of abortion storms and high mortality rates in young ruminants, particularly sheep [5]. Infection in humans causes a self-limiting febrile disease, but severe complications occur in a small percentage of cases [6].

The genome of the membrane-enveloped virus is composed of three negative-sense RNA segments: the S-segment (1690 nt) encoding the N and NSs proteins, the M-segment (3885 nt) encoding the NSm, 78-kDa, Gn and Gc proteins and the L-segment (6404 nt) encoding the L protein (RNA-dependent RNA polymerase) [7]. Grobbelaar et al. [8] described 15 distinct RVFV lineages (A–O), of which eight have been detected in South Africa. The first outbreak in South Africa was recognized in 1951 in the western Free State, southwestern Transvaal (North West Province) and the Northern Cape during late summer [9]; the single existing isolate belonged to Lineage O [8]. Two other major RVF outbreaks occurred in South Africa in 1974–1976 (Lineage L) in the Northern Cape Province, southern Orange Free State, western parts of the Transvaal and the Eshowe area in KwaZulu-Natal (KZN) and in 2008–2011, affecting almost all provinces [10]. The latter involved Lineage C in the eastern parts (2008–2009) and Lineage H in the central and western parts (2009–2011) of the country [8].

Early transmission studies in South Africa showed that *Culex* (*Culex*) *theileri*, *Culex*. (*Cux*.) *zombaensis*, *Culex*. (*Cux*.) *neavei* and *Eretmapodites quinquevittatus* were able to transmit RVFV successfully, while *Anopheles* (*Anopheles*) *coustani*, *Aedes* (*Neomelaniconion*) *mcintoshi* and *Aedes* (*Neo*.) *circumluteolus* failed to transmit the virus [11]. Transovarial transmission was suggested when RVFV was isolated in *Ae*. *mcintoshi* adults raised from larvae from an artificially flooded dambo (shallow wetland) in Kenya [12]. In a later study *Ae*. *mcintoshi* and *Ae*. *circumluteolus* demonstrated competency as RVFV vectors when adult mosquitoes, infected at the larval stage, were able to transmit the virus [13].

During an outbreak in Egypt (1977–1978), RVFV was isolated from indoor-resting *Culex* (*Cux.*) *pipiens* [14]. The transmitting capability of the virus was then tested under laboratory conditions, and it was confirmed that this species in the Nile Delta and Valley can be a vector for RVFV [14]. Isolations of RVFV were made in West and Central Africa from *Aedes* (*Aedimorphus*) *dalzieli, Aedes* (*Adm.*) *ochraceus, Aedes* (*Adm.*) *vexans, Aedes* (*Adm.*) *cumminsii, Aedes* (*Diceromyia*) *furcifer, Aedes* (*Neo.*) *palpalis, Culex* (*Cux*.) *antennatus* and *Mansonia africana* [15]), and it was isolated for the first time from *Culex* (*Cux.*) *poicilipes* in Mauritania in 2000 [16].

The mechanism for maintenance of the virus during long inter-epidemic periods (IEP) and whether vertebrate reservoirs are involved are still uncertain. Transovarial transmission of the virus in floodwater-breeding *Aedes* spp. mosquitoes is currently regarded as the primary maintenance mechanism [12], along with low-level circulation between vectors and hosts during IEPs. However, the identity of the important vector species in many areas is poorly known.

The Maputaland coastal plain in northern KZN, adjacent to the Mozambique border, has a tropical climate characterized by warm, dry winters and hot, wet summers. Although RVFV was isolated from *Ae. circumluteolus* in the area in 1955 [17], no further reports of viral circulation or cases of RVF have been reported from the area. Recently, a high rate of seroconversion to RVFV was found in cattle and goats [18], and a high seroprevalence was discovered in wild ruminants [19], suggesting that RVFV was endemic in this area of KZN, despite the absence of reported outbreaks. A recent outbreak in goats in 2014 due to a Lineage C RVFV was reported in Mozambique, ±100 km north of the study area [20].

Concurrently with the abovementioned serological study in northern KZN [18,19], mosquitoes were regularly trapped for 18 months in order to identify potential vectors in this area of endemic RVFV circulation. This paper reports the detection, sequencing and phylogenetic analysis of RVFV from a pool of *Aedes* (*Aedimorphus*) *durbanensis* collected during the study.

## 2. Materials and Methods

During 2017–2018, 34,847 mosquitoes were collected at three sites (Supplementary Table S1) in tropical north-eastern KZN, South Africa, as part of a study to investigate the epidemiology of RVF [18,19]. Mosquitoes were trapped over a period of 18 months using modified CO_2_-baited Shannon tent traps placed overnight for a minimum of 12 h. Mosquitoes were morphologically identified to species using keys [21,22] and pooled (*n* ≤ 50) by species, site and date.

Pools were homogenized in 1 mL of phosphate-buffered saline (Merck, Modderfontein, South Africa) using 2 mL homogenizing tubes (Anatech, Johannesburg, South Africa) in a Precellys 24-bead mill homogenizer (Anatech) at 6000 rpm for 1 min and centrifuged in an Eppendorf 5430 at 300 m rpm for 3 min at room temperature. The samples were stored at −80 °C until further processing. Nucleic acid was extracted from homogenized mosquito pools using the MagMAX total nucleic acid isolation kit (Applied Biosystems, Waltham, MA, USA), and extracts were screened for RVFV using real-time reverse-transcription PCR (RT-PCR) [23].

Of 627 pools screened, 105 contained *Ae. durbanensis* (Supplementary Table S1), which was very common in the study area during late summer, between February and April. One pool, comprising only unfed female *Ae. durbanensis* caught in March 2017 at Mpala, KZN, tested qPCR-positive for RVFV, but Sanger sequencing was unsuccessful. Virus isolation was attempted by intracerebral inoculation of suckling mice (NIH strain) with the RVFV qPCR-positive mosquito homogenate [24]. For this procedure the relevant animal ethics clearance was obtained from the National Institute for Communicable Diseases Animal Ethics Committee (AEC126-11_2019). Virus isolation was unsuccessful, likely due to low virus titre in the source material.

**Figure 1:**
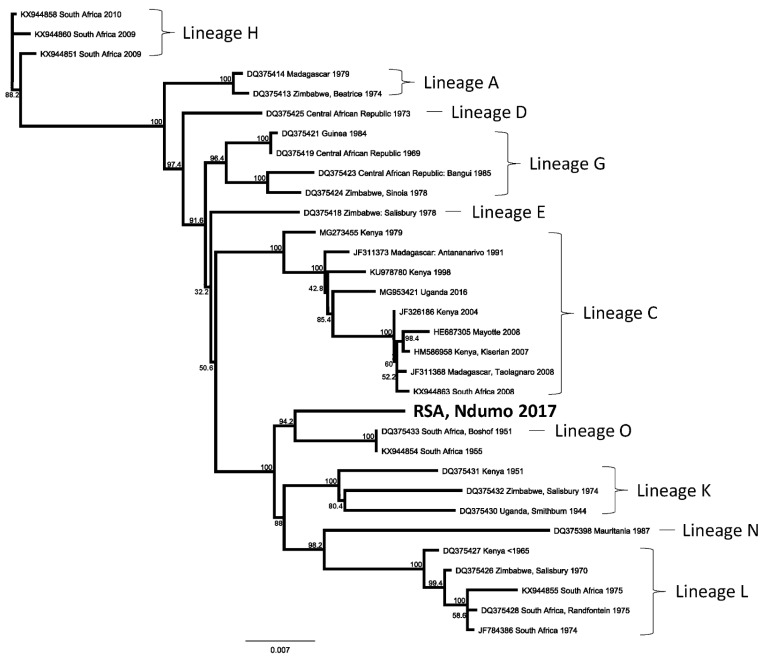
Maximum likelihood phylogenetic tree indicating the genetic relationship of the partial L segment of Rift Valley fever virus (3555 nt) detected in South Africa, 2017 (RSA_Ndumo_2017), compared to the same gene region of other sequences from GenBank, presented in a phylogram with 500 bootstrap replicates to estimate branch support. The indicated lineages follow the classification by Grobbelaar et al. [8] of the same isolates based on a 490-nt section of the Gn glycoprotein gene on the M segment. The scale bar indicates genetic distance (substitutions per site) calculated using the Tamura–Nei method.

Total nucleic acid was then extracted from the last remaining 100 μL of the *Ae. durbanensis* mosquito homogenate using the Biomeme M1 Sample Prep Cartridge Kit (Biomeme, Philadelphia, PA, USA). The presence of RVFV RNA was verified using primers/probes designed from sequence data of the L- and M-segments of the virus [25] and adapted to the Franklin Real-Time PCR Thermocycler (Biomeme, Philadelphia, PA, USA). The sample was then sequenced using a targeted PCR sequencing approach [26]. A sequence library was prepared using a cDNA-PCR sequencing Kit (Qiagen, Germantown, MD, USA) with 15 cycles (Oxford Nanopore Technologies, Oxford, UK) and sequenced using a MinION (Oxford Nanopore Technologies, Oxford, UK). Minion/ONT sequencing yielded 5502 sequencing reads. Assembly yielded 5 overlapping contigs consisting of a total of 14 long reads. Although small fragments of all three genome segments were detected, only the L segment had sufficient quality (number and length of reads) for phylogenetic analysis. The data for RVFV were assembled after trimming, mapped and the consensus sequences aligned to applicable reference strains (Supplementary Table S2). Aligned sequences were exported to Geneious version 10.2.3, realigned using ClustalW, and a maximum likelihood tree was constructed using PhyML (Figure 1).

## 3. Results

*Aedes durbanensis* was by far the most numerous *Aedes* sp. caught over the entire study period, comprising 60.4% of *Aedes* spp. and 11.7% of all mosquitoes caught (Supplementary Table S1). It was detected mainly between February and April, reaching a peak during March, when it comprised 69.3% of *Aedes* spp. and 15.6% of all mosquitoes caught.

The 105 pools of unfed *Ae. durbanensis* that were screened contained 4077 mosquitoes, representing 3157 (83 pools) from 2017 and 920 (22 pools) from 2018. One RVFV-positive pool was found out of 62 pools, containing 3018 mosquitoes, trapped in March 2017, resulting in a minimum infection rate (MIR) in *Ae. durbanensis* of 0.03% in March 2017 and 0.02% over the entire study period.

The RVFV identified in this study (RSA, Ndumo 2017) was clustered with a Lineage O virus isolated in 1951 from a sheep in the Free State Province in central South Africa, approximately 800 km from the collection site, as well as with another isolate from 1955, of uncertain origin but likely also from the same area (Figure 1). The partial RSA, Ndumo 2017 L-segment sequence shared 98.01% identity on a nucleotide level and 99.13% identity on an amino acid level with its closest relative, the 1951 Free State isolate. The accession number of the isolate (Partial Segment L, 3555 nt) in GenBank is MW183126.

## 4. Discussion

Multiple arboviruses were isolated in the 1950s and 1960s, including RVFV in 1955, from various mosquito species in the vicinity of the study area in far north-eastern KZN [17,27,28]; however, no further RVFV isolations have been made in that area since then, nor have any cases of RVF been reported. The isolation of the virus in this study confirms that RVFV is circulating in the area more than 60 years later. The clustering of the recent isolate with two isolates from central South Africa in 1951 and 1955 indicates the possibility that the movement of an infected animal from KZN, an endemic area, to the Free State, an area unsuitable for year-round RVFV circulation but with large numbers of susceptible sheep, might have initiated the first large outbreak in South Africa in 1950–1951.

The two RVFV isolates from KZN in 1955 were obtained from *Ae. circumluteolus* at Simbu Pan, a shallow, seasonally flooded wetland on the floodplain of the Pongolo River [17], about 20 km from the current collection site, and represented two different lineages, I and M, based on a 490-nt M-segment sequence [8]; unfortunately, L-segment sequences from those isolates were not available for inclusion in the current analysis. The current isolate was not closely related to other more recent isolates from eastern South Africa (Lineage C), the isolate responsible for the most recent large outbreak in South Africa in 2010–2011 (Lineage H) or the small outbreak in 2014 in southern Mozambique (Lineage C) [20]. Based on previous isolations in eastern South Africa, Lineage C RVFV was the most likely virus expected in the area, but since this was not the case it further suggests that there is more than one lineage circulating in the area. This was the case in Kenya during 2006–2007, when multiple lineages including B, C, K and L circulated in parallel in the area [8,29]. The same situation occurred in Zimbabwe in 1978, where the co-circulation of multiple strains was suggested in the area around Harare [8,30].

Although *Ae. durbanensis* was implicated in a disease outbreak where sheep presented with RVF-like symptoms in Kenya in 1937 [31], RVFV has not previously been detected in the species, and its vector competence for RVFV has not been established. However, it is closely related to *Ae. vexans*, an important RVFV vector in West Africa [15]. Considering that *Ae. durbanensis* was found in our study to be seasonally extremely numerous in northern KZN, and that the isolation was from unfed mosquitoes, it seems likely that it could serve as a vector in this area. The low MIR of RVFV in the mosquitoes tested (0.03%) is not inconsistent with detection rates in vectors reported elsewhere, even during outbreaks, which is generally less than 0.1% [32,33,34]. Therefore, the RVFV vector competence of *Ae. durbanensis* and its potential role in the epidemiology of RVF should be investigated.

Due to its zoonotic and vectorborne nature, its socioeconomic impact and complex eco-epidemiology, RVF requires an interdisciplinary “One Health” approach for effective surveillance, investigation, prevention and control [35]. Vector studies such as this are therefore important in order to improve our knowledge of the determinants of RVFV circulation and emergence. Further research is required into the eco-epidemiology and potential socioeconomic impact of RVFV in the eastern tropical coastal region of southern Africa.

## 5. Conclusions

Based on the detection of RVFV in unfed *Ae. durbanensis* and the seasonal abundance of the species, it may be an important vector of RVFV in northern KZN, South Africa. However, very little is known about its competence as a vector and its role in the epidemiology of RVF. This study demonstrated that further work is required to determine the vectors responsible for RVFV circulation in the eastern tropical coastal region of southern Africa, as well as the potential of the spread of RVFV to initiate outbreaks in other areas.

## Data Availability

The datasets generated during and/or analyzed during the current study can be find in the main text and the Supplementary Materials. The accession number of the isolate (Partial Segment L, 3555 nt) in GenBank is MW183126.

## Supplementary Materials

The following supporting information can be downloaded at: https://www.mdpi.com/article/10.3390/pathogens11020125/s1: Table S1. Mosquito species and the total number of mosquitoes collected; Table S2. Rift Valley fever virus isolates used as reference strains and for construction of the phylogenetic tree.
