# Supplementary material for: Detection of Rift Valley Fever Virus in Aedes (Aedimorphus) durbanensis, South Africa
Source: Pathogens. 2022 Jan 21;11(2):125. doi: 10.3390/pathogens11020125 (PMC8879006; doi:10.3390/pathogens11020125)
Supplement: Supplementary file 1 [file pathogens-11-00125-s001.zip › pathogens-1503534-supplementary/Table S1.pdf]

Supplementary Table S1. Mosquito species and the total numbers of mosquitoes collected over the study period (2017-2018) at Mpala (-26.941S, 32.218E), Bumbe (-26.997S, 32.301E) and Makhana (-27.005S, 32.289E).

Species	Total	%
Anopheles (*Ano.*) *coustani*	250	0.7%
An. (*Ano.*) *ziemanni*	63	0.2%
An. (*Cel.*) *funestus* grp	77	0.2%
An. (*Cel.*) *gambiae*	269	0.8%
An. (*Cel.*) *maculipalpis*	23	0.1%
An. (*Cel.*) *pharoensis*	172	0.5%
An. (*Cel.*) *pretoriensis*	58	0.2%
An. (*Cel.*) *rufipes*	170	0.5%
An. (*Cel.*) *squamosus*	284	0.8%
An. (*Cel.*) *theileri*	9	<0.1%
Anopheles spp.	112	0.3%
Aedes (*Adm.*) *albocephalus*	18	0.1%
Ae. (*Adm.) argenteopunctatus* grp	5	<0.1%
Ae. (*Adm.*) *cumminsii*	112	0.3%
Ae. (*Adm.*) *dentatus/leesoni*	1	<0.1%
Ae. (*Adm.*) *durbanensis*	4083	11.7%
Ae. (*Adm.) fowleri*	146	0.4%
Ae. (*Adm.*) *hirsutus*	2	<0.1%
Ae. (*Adm.*) *leesoni* grp	19	0.1%
Ae. (*Adm.*) *ochraceus*	80	0.2%
Ae. (*Adm.*) *subdentatus*	5	<0.1%
Ae. (*Adm.*) *veeniae*	1	<0.1%
Ae. (*Muc.*) *sudanensis*	179	0.5%
Ae. (*Neo.*) *aurovenatus*	1	<0.1%
Ae. (*Neo.*) *circumluteolus*	1105	3.2%
Ae. (*Neo.*) *mcintoshi*	822	2.4%
Ae. (*Ste.*) *aegypti*	13	<0.1%
Ae. (*Ste.*) *metallicus*	25	0.1%
Aedes spp.	147	0.4%
Coquillettidia chrysosoma	8	<0.1%
Cq. metallica	1	<0.1%
Culex (*Cux.*) *annulioris*	22	0.1%
Cx. (*Cux.*) *antennatus*	9980	28.6%
Cx. (*Cux.*) *decens*	1	<0.1%
Cx. (*Cux.*) *ethiopicus*	4	<0.1%
Cx. (*Cux.*) *neavei*	3427	9.8%

Cx. (*Cux.*) *perfuscus*	168	0.5%
Cx. (*Cux.*) *pipiens*	169	0.5%
Cx. (*Cux.*) *poicilipes*	319	0.9%
Cx. (*Cux.*) *simpsoni*	9	<0.1%
Cx. (*Cux.*) *sitiens*	1	<0.1%
Cx. (*Cux.*) *thalassius*	7	<0.1%
Cx. (*Cux.*) *theileri*	3	<0.1%
Cx. (*Cux.*) *tritaeniorhynchus*	10959	31.4%
Cx. (*Cux.*) *vansomereni*	2	<0.1%
Cx. (*Cux.*) *zombaensis*	191	0.5%
Cx. (*Lut.*) *tigripes*	5	<0.1%
Culex spp.	133	0.4%
Mansonia (*Man.*) *africana*	529	1.5%
Ma. (*Man.*) *uniformis*	656	1.9%
Mimomyia plumosa	1	<0.1%
Uranotaenia sp.	1	<0.1%
