# Supplementary material for: Detection of Rift Valley Fever Virus in Aedes (Aedimorphus) durbanensis, South Africa
Source: Pathogens. 2022 Jan 21;11(2):125. doi: 10.3390/pathogens11020125 (PMC8879006; doi:10.3390/pathogens11020125)
Supplement: Supplementary file 1 [file pathogens-11-00125-s001.zip › pathogens-1503534-supplementary/Table S2.pdf]

Supplementary Table S2. Rift Valley fever virus isolates used as reference strains and for construction of the phylogenetic tree.

**Rift Valley fever virus – reference strain: KX096938 (Kenya-128b-15)**				
**Genbank Accession no.**	**Isolate no.**	**Year**	**Location**	**Source**
DQ375398	OS-1/87	1987	Mauritania	Human
DQ375413	2250/74	1974	Zimbabwe, Beatrice	Bovine foetus
DQ375414	MgH824/79	1979	Madagascar	Human
DQ375418	1260/78	1978	Zimbabwe, Salisbury	Bovine foetus
DQ375419	Zinga67	1967	Central African Republic	Human
DQ375421	ANK-6087/74	1974	Guinea	*Micropterus pusillus*
DQ375423	CAR-R1622/85	1985	Central African Republic, Bangui	Human
DQ375424	1853/78	1978	Zimbabwe, Sinoia	Bovine foetus
DQ375425	73HB1230	1973	Central African Republic	Human
DQ375426	730/70	1970	Zimbabwe, Salisbury	Bovine foetus
DQ375427	KenyaIB8/65	1956	Kenya	Bovine
DQ375428	SA-75	1975	South Africa, Randfontein	Human
DQ375430	Smithburn	1944	Uganda	Derived from RVFV Entebbe
DQ375431	Kenya57/51	1951	Kenya	Ovine
DQ375432	2373/74	1974	Zimbabwe, Salisbury	Bovine foetus
DQ375432	SA-51	2007	South Africa, Boshoff	Ovine
HE687305	099/2008	2008	Mayotte	Human
HM586958	KEN/Bar-035/07	2007	Kenya, Kiserian, 2007	Human
JF311368	0212/08	2008	Madagascar, Taolagnaro	Human
JF311373	ZF-06/1991	1991	Madagascar, Antananarivo	Bovine
JF784386	35/74	1974	South Africa	Ovine
JF326186	23/2004	2004	Kenya	Human
KU978780	Ken 523/98	1998	Kenya	Human
KX944851	M127/09	2009	South Africa	Bovine
KX944854	M1955	1955	South Africa	Unknown
KX944855	M1975Bov	1975	South Africa	Bovine
KX944858	M25/10	2010	South Africa	Ovine

KX944860	M260/09	2009	South Africa	Bovine
KX944863	M37/08	2008	South Africa, Hoedspruit	Buffalo calf
MG273455	Kenya 90058 (B-691)	1979	Kenya	Bovine
MG953421	201601502	2016	Uganda	Human
